# Differences in lower limb muscle activation between global and selective instability devices in single-leg stance in healthy active subjects

**DOI:** 10.7717/peerj.13317

**Published:** 2022-04-18

**Authors:** Mariana Sánchez-Barbadora, Alba Cuerda-Del Pino, Javier González-Rosalén, Noemi Moreno-Segura, Adrian Escriche-Escuder, Rodrigo Martín-San Agustín

**Affiliations:** 1 Physiotherapy, University of Valencia, Valencia, Spain; 2 Physiotherapy, University of Malaga, Malaga, Spain; 3 Faculty of Health Sciences, Universidad Internacional de Valencia—VIU, Valencia, Spain

**Keywords:** Electromyography, Peroneus, Ankle, Balance

## Abstract

**Background:**

Balance and strength training are frequent strategies to address lower limb injuries, including ankle injuries, which are usually performed in single-leg stance on global instability devices, producing generalized muscular activation of the lower limb. In this context, new specific instability devices arise from the need to selectively work the ankle, specifically the peroneus longus. This study aimed to compare the EMG muscle activation of the peroneus longus, as well as other lower limbs muscles, in a single-leg stance on different balance training devices (BOSU, wobble board, power board, and Blackboard) in standing or squatting positions.

**Methods:**

Twenty healthy recreationally trained subjects participated in the study. Subjects performed three repetitions of 15 s (one for familiarization and two for measurement) in standing and squatting positions on the floor, BOSU, wobble board, power board, and Blackboard. Surface electromyography (EMG) was used to record activity of the peroneus longus, soleus, gastrocnemius medialis, tibialis anterior, rectus femoris, and gluteus maximus.

**Results:**

The main outcome was that no differences were found for the peroneus longus normalized EMG, neither between devices (*p* = 0.09) nor between conditions (*p* = 0.11), nor in the interaction between them (*p* = 0.16). For the normalized EMG of the other muscles, there were multiple differences between devices and conditions. Of the devices studied, the Blackboard was the one that implied a lower activation of the lower limb muscles and a lower degree of instability, activating the peroneus longus similarly to global instability devices. The BOSU and wobble board achieved high levels of EMG muscle activation for most muscles of the lower limbs. Therefore, they should be considered as potential devices for work in highly unstable conditions or when high activation levels are sought.

## Introduction

Ankle injury is among the most common pathologies treated in primary care and emergency services, involving approximately 25% of all injuries of the musculoskeletal system and 50% of all sports-related injuries (Czajka et al., 2014). This injury, characterized by excessive stretching or tearing of the ankle ligaments, is usually acute. However, some studies quantify the incidence of residual symptoms after acute ankle sprain at between 40 and 50% (Gerber et al., 1998; Konradsen et al., 2002; Van Rijn et al., 2008; Hubbard-Turner & Turner, 2015). Among these residual symptoms, we can find lateral ankle pain with long evolution times, caused by ankle instability (better known as Chronic Ankle Instability, CAI) or other differential causes (Gerber et al., 1998; Konradsen et al., 2002; Van Rijn et al., 2008; Hubbard-Turner & Turner, 2015).

In this regard, EMG muscle activation of the peroneus longus (Pero-L) and brevis, in particular, has shown to be delayed following sprains and in the presence of fatigue (Keles et al., 2014; Rodrigues, Soares & Tomazini, 2019). Likewise, changes are found in the morphology and pennation angle of Pero-L in chronic ankle instability (Yoshida & Suzuki, 2020). According to the findings of a previous study, Pero-L plays an important role in the eversion and plantarflexion of the ankle (Mendez-Rebolledo et al., 2021). Therefore, a tailored Pero-L approach may be essential to manage and prevent ankle injury (Han & Ricard, 2011; Thompson et al., 2018). In turn, since ankle inversion/eversion is accompanied by hip axial rotation during single-leg stance (primarily characterized by inter-joint coordination) (Liu et al., 2012), training of other muscles involved in both ankle and hip movements is also essential.

Considering the previous, exercise therapy is one of the main approaches used to treat and prevent the recurrence of ankle sprains (Doherty et al., 2017) and CAI (McKeon & Hertel, 2008). In particular, balance and strength training performed in single-leg stance have proven to be effective in improving proprioception, neuromuscular control, and sensorimotor system (Gauffin, Tropp & Odenrick, 1988; Lazarou et al., 2018; Hall et al., 2018; Vuurberg et al., 2018). Balance training is frequently approached through training in conditions of instability. This training is characterized by exercises performed with devices or postures challenging postural control. Unlike traditional strength training, this approach has previously shown to facilitate the recruitment of muscle fibers for maintaining body stability. In addition, a fundamental aspect of balance training is the progression in the exercises using, for example, platforms with different levels of stability (Borreani et al., 2014).

The most popular balance training methods include devices such as BOSU, wobble board (WB), or power board (PB) (Saeterbakken & Fimland, 2013; Strøm et al., 2016), which have traditionally been used as exercise therapy for ankle rehabilitation and injury prevention. These global instability devices have proven to be very demanding in the inversion-eversion movement (Strøm et al., 2016). However, they do not allow their modification to progress or adapt to the difficulty of the exercise, and the structure of the foot to be worked cannot be selectively determined. Therefore, they produce a generalized muscle activation of the entire lower limb (Silva et al., 2016). This fact contrasts with the effect of selective devices such as the Mini Stability Trainer (Ludwig ARTZT GmbH, Germany), which produces different muscle activations of the lower limb muscles depending on whether the forefoot or rearfoot is destabilized (higher activation of most of the lower limb muscles when the forefoot is destabilized) (Alfuth & Gomoll, 2018).

With the intention of directly addressing the application of forefoot or rearfoot instability, additional selective balance training alternatives have been developed, also finding differences in the activation of the muscles of the lower limbs using specific forefoot or rearfoot destabilization procedures (*e.g.*, Exercise Sandals (Michell et al., 2006), Ankle-Destabilization Boot and Ankle-Destabilization Sandal (Forestier & Toschi, 2005; Donovan, Hart & Hertel, 2014; Donovan, Hart & Hertel, 2015), or StepRight Stability Trainer (Bouillon et al., 2019)). Most of these devices focus directly on the ankle joint, specifically on the Pero-L (Forestier & Toschi, 2005; Donovan, Hart & Hertel, 2014; Donovan, Hart & Hertel, 2015; Bouillon et al., 2019). However, some of them have the disadvantage of being excessively complex or bulky, not configurable to progress in the rehabilitation process, or not easily portable. Others, such as the device investigated by Alfuth & Gomoll (2018), have improved configurability (*e.g.*, select instability direction) and portability aspects but remain limited in terms of progression in complexity. In this sense, a recently developed device known as Blackboard Training (BB) has been designed in which instability direction and degree settings can be adjusted by the user. This characteristic could favor the progression in complexity and activate specific ankle muscles.

In this context, the study of how selective and standard devices specifically activate the muscles related to the kinematics of the ankle is required. According to a previous study, comparisons of the surface electromyography (EMG) signal across different exercises may provide insight into muscular force production, despite some limitations (Vigotsky et al., 2017). In turn, in balance training, muscle activity has been used as an indicator of the intensity produced by the instability of the device used (Strøm et al., 2016). Thus, previous authors have suggested the importance for a clinician of distinguishing between the different devices concerning perturbation potential (*i.e.,* ability to produce kinematic alterations through instability) and intensity (Strøm et al., 2016) or between different positions that affect these as well (*e.g.*, standing or squat position) (Wahl & Behm, 2008).

However, although some of these selective instability devices have been analyzed in terms of the EMG muscle activation produced (Forestier & Toschi, 2005; Donovan, Hart & Hertel, 2014; Donovan, Hart & Hertel, 2015; Alfuth & Gomoll, 2018; Bouillon et al., 2019), to date there are no studies comparing the EMG muscle activity produced by one of them against multiple global instability devices.

The main hypothesis of this study is that balance training using a selective device as BB results in differences in the activity of lower limb muscles compared with global instability devices, since these are devices with different perturbation potential. In this sense, it is hypothesized that BB configured for anteversion instability of the rearfoot produces similar Pero-L muscle EMG activation that global instability devices but lower in other lower limb muscles.

Therefore, the main aim of this study was to compare the EMG muscle activation of the Pero-L as well as other lower limbs muscles in a single-leg stance on different balance training devices (BOSU, WB, PB, and BB) in standing or squatting positions.

## Materials & Methods

### Study design

In this observational study, the activation of the Pero-L, soleus, gastrocnemius medialis (Gastr-M), tibialis anterior (Tib-A), rectus femoris (Rect-F), and gluteus maximus (Glut-M) muscles was measured in a single day of testing. Performance conditions registered were standing and squatting positions on the floor, BOSU, WB, PB, and BB. Both standing and squat positions were proposed to compare the level of muscle activation between four instability systems.

### Subjects

Twenty healthy recreationally trained participants, ten males (mean age: 23.40 ± 2.91 years; body mass: 79.80 ± 8.42 kg; stature: 177.9 ± 3.07 cm; weekly physical activity: 359.00 ± 211.05 min) and ten females (mean age: 22.30 ± 1.06 years; body mass: 57.20 ± 11.17 kg; stature: 164.00 ± 6.02 cm; weekly physical activity: 282.00 ± 168.05 min), were recruited through a call for volunteers by the Faculty of Physiotherapy at the University of Valencia. All participants performed exercise; three times per week and practiced activities such as running, swimming, cycling, or general strength training (Martín-San Agustín et al., 2018). For inclusion, participants had to be between 18 and 30 years old, have no history of lower limb injury or pain during the year preceding the study and had to perform at least 90 min of physical activity per week. The established exclusion criteria were the contraindication of the use of adhesive electrodes either due to injury or adhesive allergy, to have previously participated in some balance improvement or lower limb proprioception program, or presenting any known balance disorder, such as vertigo, or vestibular or central nervous system alterations. All participants had to provide written informed consent and complete a basic information form prior to data collection, which included demographic (age and sex) and anthropometric measures (height and weight). The experimental protocol was approved by the Ethics Committee of the University of Valencia (Spain) (H1544554364247).

### Procedures

**Figure 1:**
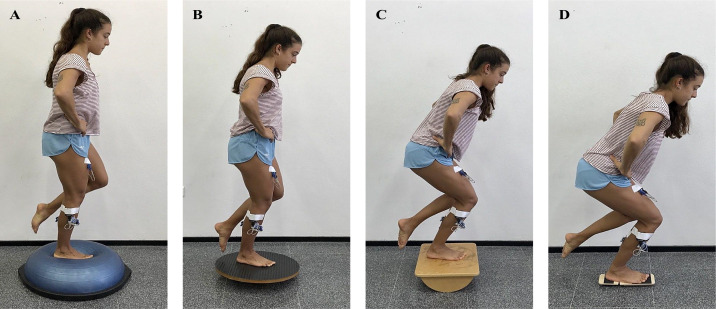
Different conditions of measurement of the EMG. Single-leg standing on BOSU (A) and on WB (B) and single-leg squat on PB (C) and on BB (D).

EMG muscle activity was registered with the participants first on the floor and then on the various devices. All measurements were made in both standing and squatting positions, as previous authors have suggested differences in activation for leg and trunk musculature between positions (Wahl & Behm, 2008). The order of measurements in standing and squatting positions, as well as that of the different devices, was randomized. Participants performed three single-leg standings (0° of knee extension) and three single-leg squats (60° of knee flexion) trials on the dominant leg using one stable control (rigid floor) and four unstable devices (Fig. 1). While the initial range of motion (0° or 60°) was established with a goniometer, the subjects could make small variations of the range of motion to balance against instability during the maintenance of the single-leg standing or squat.

Devices used were BOSU, with the bladder side up, WB, PB, oriented in the sagittal plane, and BB, with its instability in the rearfoot. Previously, device instability requirements have been described regarding the number of unstable dimensions and the magnitude of contact with the floor. While PB was only unstable in 1 dimension (front–back position), BOSU and WB were unstable in 2 dimensions. In turn, BOSU has a larger support base than the WB, so theoretically, it would be considered less unstable than the WB (Saeterbakken & Fimland, 2013).

**Figure 2:**
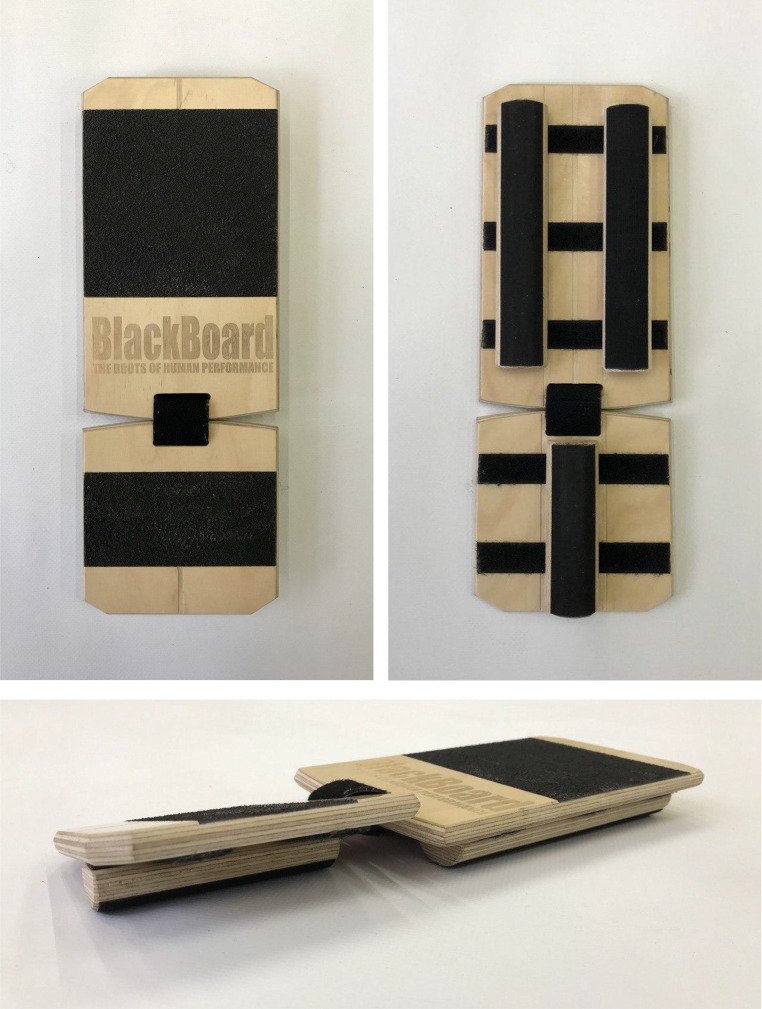
Selective instability device: Blackboard Trainer.

Moreover, the BB is a device designed for single-leg stability training, consisting of two wooden boards connected by a strap. Its base has a Velcro surface on which half-cylinder-shaped wooden slats can be freely placed (Fig. 2). In this study, the slats were positioned so that the forefoot was fixed and the hindfoot remained unstable, with the slat placed longitudinally in the center of the board. Thus, although BB solo was only unstable in one dimension, the support base was the lowest of all devices. The foot was placed central to the platform for the first three unstable devices. For the Blackboard, the tuberosity of the fifth metatarsal was taken as a reference, which coincided with the spacing between the two wooden boards.

Participants were barefoot with their eyes open as this is the most common methodology in similar studies (Laudner & Koschnitzky, 2010; Harput et al., 2013; Alfuth & Gomoll, 2018). Initially, the knee of the supporting leg was straight for the standing measurements, and at 60° of flexion measured with a goniometer for the squat measurements (Wahl & Behm, 2008), with their hands placed on their hips. After a trial to become familiar with each condition and taking the control measurements on the rigid floor, participants performed two repetitions of 15 s, with 2 min rest between them, on each device (Wahl & Behm, 2008). The trial was considered unacceptable if the participant left the device, displaced it from its usual location during the measurement or touched the floor with the contralateral foot (Alfuth & Gomoll, 2018).

**Figure 3:**
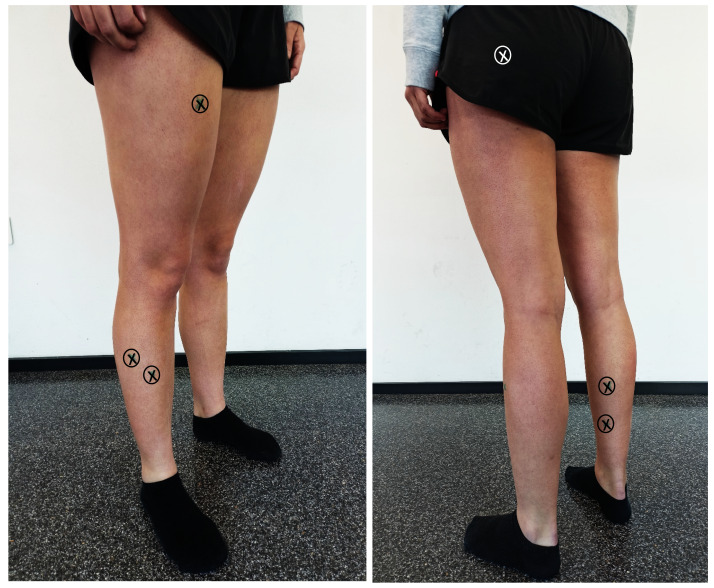
Electrode placement. The circles with a cross indicate the place of placement of the electrodes.

Surface EMG was used to record the activity of the Pero-L, soleus, Gastr-M, Tib-A, Rect-F, and Glut-M by MuscleLab 4020e (Ergotest Technology, Stathelle, Norway). The skin was shaved and cleaned with alcohol. The recommendations of the SENIAM project (Hermens et al., 2000) were followed to apply the pre-gelled Ag/AgCl EMG electrodes (BlueSensor N; Ambu Ballerup, Denmark). After placing them (Fig. 3 shows the electrode placement), the examiner performed a manual muscle testing procedure by palpating the muscle belly and verifying accurate electrode location. The raw EMG signals were processed as previously described in Van den Tillaar & Larsen (2020). Specifically, raw EMG signals were sampled at 1,000 Hz. Then, the signals were high pass and low pass filtered with a cutoff frequency of 20 Hz and 500 Hz, and subsequently rectified, integrated, and converted to root-mean-square (RMS) signals using a hardware circuit network (frequency response 450 kHz, averaging constant 12 ms, total error ± 0.5%). The filtered EMG signal was normalized to the muscle activity obtained from the floor trial (Silva et al., 2016).

Of the total 15 s collected, the initial and final 5-second periods of normalized EMG (nEMG) were discarded to minimize postural adjustments or fatigue. Thus, nEMG amplitude over the central 5-second period was calculated for each trial (Wahl & Behm, 2008). nEMG mean of the two attempts in each condition was calculated and used for the subsequent comparison.

### Statistical analysis

SPSS Statistics 25 (IBM Corporation, Chicago, Illinois, USA) was used to perform all statistical analyses. Descriptive statistics of each variable (including means, 95% confidence intervals, and standard deviations) were defined. To determine differences in Pero-L nEMG for all different unstable surfaces, as well as in the rest of the muscles, repeated-measures split-plot analyses of variance (ANOVAs) were used.

For the nEMG of Pero-L, as well as for the other muscles, a two-way repeated-measures ANOVA was used to determine differences between devices, conditions, and the interaction between devices and conditions, in a similar way to what was done in previous studies (Martín-San Agustín et al., 2021). Additionally, in the case of detecting a significant main effect, post hoc t tests with Bonferroni corrections were applied to establish the identity of the differences. The level of significance was set at *p* < 0.05. The effect size was assessed using *η*^2^ (partial-Eta squared) where 0.01 <*η*^2^ <0.06 was considered a small effect, 0.06 <*η*^2^ <0.14 a medium effect, while *η*^2^ >0.14 was considered a large effect (Cohen, 1977).

## Results

Table 1 shows the absolute EMG values (µV) for each muscle by condition on the floor.

**Table 1:** Mean EMG (µV) of the six muscles, 95% confidence interval (CI), and standard deviation (SD) in stable surface (floor) during standing and squat monopodal position.

Condition	Peroneus longus	Soleus	Gastrocnemius medialis	Tibialis anterior	Rectus femoris	Gluteus maximus
	Mean; 95% CI (SD)	Mean; 95% CI (SD)	Mean; 95% CI (SD)	Mean; 95% CI (SD)	Mean; 95% CI (SD)	Mean; 95% CI (SD)
Standing	63.65; 46.81–80.49 (8.05)	32.43; 24.68–40.17 (3.70)	49.23; 37.26–61.19 (5.72)	51.43; 37.18–65.67 (6.81)	8.25; 5.09–11.41 (1.51)	7.28; 5.06–9.49 (1.06)
Squat	71.28; 52.48–90.07 (8.98)	42.05; 30.96–53.14 (5.30)	24.38; 16.67–32.08 (3.68)	76.10; 53.80–98.40 (10.65)	39.23; 21.63–56.82 (8.41)	18.98; 13.65–24.30 (2.54)

**Figure 4:**
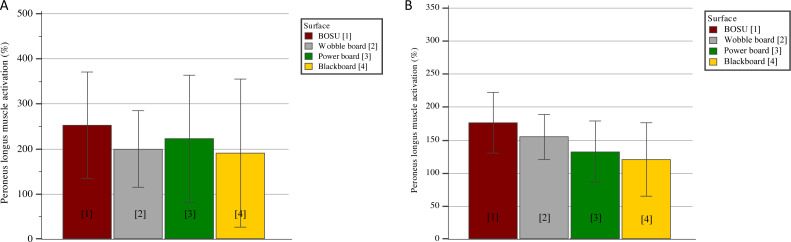
Peroneus longus muscle activation (nEMG) in different unstable surfaces in (A) standing and (B) squat monopodal position (mean and 95% CI).

Figure 4 and Table 2 show the nEMGs for Pero-L and the other muscles, respectively. Regarding the comparisons for the Pero-L nEMG (Fig. 4), there were no differences either between devices (*p* = 0.09; *η*^2^ = 0.12) or between conditions (*p* = 0.11; *η*^2^ = 0.12), nor in the interaction between them (*p* = 0.16; *η*^2^ = 0.09).

**Table 2:** Difference in nEMG of each muscle by device and condition*.

Muscle	Condition	BOSU (%)	WOBBLE BOARD (%)	POWER BOARD (%)	BLACKBOARD (%)	Inter-device differences^†^
		Mean; 95% CI (SD)	Mean; 95% CI (SD)	Mean; 95% CI (SD)	Mean; 95% CI (SD)	
Gluteus maximus	Standing	456.31; 263.01–649.60 (92.35)	403.96; 256.86–551.07 (70.28)	349.01;218.30–479.72 (62.45)	186.74; 132.89–240.59 (25.73)	WB-BB
	Squat^‡^	273.95; 124.41–423.48 (71.44)	211.68; 169.80–253.56 (20.01)	196.70; 153.67–239.74 (20.56)	189.81; 135.11–244.51 (26.13)	
Rectus femoris	Standing	452.12; 295.21–609.02 (74.96)	493.65; 268.62–718.67 (107.51)	308.83; 193.32–424.34 (55.19)	165.34; 102.91–227.76 (29.82)	BO-BB; WB-BB; PB-BB
	Squat^‡^	221.00; 133.50–308.49 (41.80)	176.62; 100.54–252.70 (36.35)	149.05; 110.77–187.33 (18.29)	123.57; 88.07–159.06 (16.96)	
Tibialis anterior	Standing	294.52; 194.03–395.02 (48.01)	328.90; 180.12–477.69 (71.09)	277.54; 151.74–403.34 (60.11)	158.65; 58.75–258.55 (47.73)	BO-BB; WB-BB; PB-BB
	Squat	257.00; 153.66–360.34 (49.38)	226.28; 165.00–287.57 (29.28)	196.16; 139.01–253.31 (27.31)	137.57; 99.81–175.33 (18.04)	
Gastrocnemius medialis	Standing	205.24; 147.70–262.78 (27.49)	216.49; 134.62–298.36 (39.12)	133.85; 105.86–161.83 (13.37)	118.08; 94.13–142.03 (11.44)	BO-PB; WB-PB
	Squat	227.09; 167.64–286.54 (28.40)	252.52; 173.04–332.00 (37.97)	147.03; 90.47–203.58 (27.02)	166.99; 73.10–260.88 (44.86)	
Soleus	Standing	252.12; 184.79–319.45 (32.17)	256.24; 181.36–331.11 (35.77)	180.33; 141.11–219.55 (18.74)	143.44; 99.60–187.27 (20.94)	BO-PB; BO-BB; WB-PB; WB-BB
	Squat^‡^	204.98; 145.98–263.98 (28.19)	197.06; 134.45–259.67 (29.91)	114.83; 90.54–139.13 (11.61)	118.14; 81.50–154.78 (17.51)	

**Notes.**

*All values of devices are expressed in percent (normalized by EMG’s floor) † Abbreviations (BO = BOSU; WB: wobble board; PB = power board; BB = blackboard) indicate statistical significance (*p* ≤ 0.05). ‡ Differences between conditions (*p* ≤ 0.05). SD = standard deviation; CI = confidence interval.

For the nEMG of the remaining muscles, there were multiple differences between devices regardless of the condition (Table 2); soleus (*p* = 0.01; *η*^2^ = 0.46), Gast-M (*p* = 0.01; *η*^2^ = 0.26), Tib-A (*p* = 0.01; *η*^2^ = 0.33), Rect-F (*p* = 0.01; *η*^2^ = 0.32), and Glut-M (*p* = 0.02; *η*^2^ = 0.22), all of them associated with large effect sizes. In particular, BOSU produced a greater activation of soleus and Gast-M (*p*’s values = 0.01) than PB and of soleus, Tib-A, and Rect-F (*p*’s values = 0.01) than BB. In addition, muscle activation achieved by WB was greater than PB-based activation for soleus and Gastr-M (*p*’s values = 0.01), and greater than BB-based activation for soleus, Tib-A, Rect-F, and Glut-M (*p*’s values = 0.01). Finally, activation of Tib-A and Rect-F (*p*’s values = 0.01) was larger with PB than with BB.

In terms of comparisons between conditions (Table 2), standing position was associated with a higher activation of soleus (208.1% *versus* 158.7%; *p* = 0.01), Rect-F (354.9% *versus* 167.5%; *p* = 0.01), and Glut-M (349.0% *versus* 218.0%; *p* = 0.04) as compared to the squat position. Finally, a significant interaction between device and condition was observed for Rect-F (*p* = 0.03; *η*^2^ = 0.18) and Glut-M (*p* = 0.01; *η*^2^ = 0.22). In post hoc analysis, in Rect-F nEMG, differences were found both in standing position between BB and the other devices (lower activation in BB compared to BOSU, WB, and PB) (*p*’s range = 0.01 to 0.03) and in squat position between BB and BOSU (*p* = 0.03), with lower values for BB. Otherwise, in Glut-M nEMG, only significant differences (*p*’s values = 0.01) in standing position were found between BB and the other devices (lower activation in BB compared to BOSU, WB, and PB).

## Discussion

Our results support our initial hypothesis that BB configured for anteversion instability of the rearfoot produces a similar Pero-L activation to that obtained with global instability devices but less for other lower limb muscles. Thus, the main finding was that there were no differences between the nEMG of Pero-L between BB and BOSU, WB and PB, but, depending on the muscle, lower values were found for BB in soleus, Tib-A, Rect-F, and Glut-M than those global instability devices. Additionally, differences were found between standing or squat positions in soleus, Rect-F, and Glut-M muscle activation.

As far as we know, this is the first study comparing several global balance training devices with a selective instability device. On the one hand, the main result observed was that there are no differences in Pero-L muscle activation between devices. Accordingly, Pero-L levels of activation on the four different surfaces (BOSU, WB, PB, BB) were about 150% (squat) and 200% (standing) of muscle activation on the floor, this being consistent with the results reported for other selective (Alfuth & Gomoll, 2018) and global (Harput et al., 2013; Strøm et al., 2016) instability devices. This finding could be due to the fact that an instability involving pronosupination of the calcaneus is enough to achieve similar levels of activation to those obtained by overall balance devices.

On the other hand, activation differences were noted for other limb muscles (*e.g.*, soleus, Gastr-M, Tib-A, Rect-F, and Glut-M) between global and selective devices. Compared to PB and BB, BOSU and WB increased activation in around 100% in the lower leg muscles, the Rect-F and the Glut-M. Furthermore, BB-based activation levels in other muscles were around 100%–200% lower than those produced by global devices. The present study shows a trend whereby BOSU and WB generate greater instability than PB, which may be plausible bearing in mind the axis of movement of each device. Both BOSU and WB are multidirectional, while the PB only generates instability in one plane (sagittal in the case of this study). In addition, other studies have analyzed a different PB configuration (*e.g.*, with the instability in the coronal plane), likewise obtaining a similar Rect-F activation on PB, on BOSU and on WB (Saeterbakken & Fimland, 2013). Still, since PB destabilizes the ankle in a global movement of flexion/extension, the instability is not as specific as that achieved by BB and produces greater muscle activation of the Rect-F and Tib-A.

Despite the importance of results obtained, this study has also several limitations. First, population studied includes only healthy participants, so its results cannot be extrapolated to a pathological population. Thus, further research is needed on this device in pathological subjects. Secondly, measurements were taken in single-leg stance only, while other authors have carried out similar studies recording the muscle activation produced in the performance of various exercises (Wahl & Behm, 2008; Saeterbakken & Fimland, 2013; Harput et al., 2013). However, being the first study developed with the inclusion of the BB device, we believe that the results could be a starting point for future research work on this device, further featuring dynamic movements and interventions with different exercises. Finally, the study of a larger number of leg muscles would allow a better understanding of muscle response on a selective instability device. In the same way, the inclusion for comparison of some of the selective instability devices previously studied would be interesting.

The comparison between selective and global instability devices carried out in this study might serve as a guide in the progression of an ankle rehabilitation or injury prevention program, using muscle activity as an indicator of the degree of instability and intensity produced by the devices. As previously mentioned, an alteration caused in the rearfoot elicits a similar electromyographic response (activation) of the Pero-L while causing less activation of the remaining lower limb muscles compared to global instability devices. This could indicate that the device that implies a lower intensity for the entire lower limb is the BB without losing activation of the Pero-L. This may be desirable in the initial stages of ankle rehabilitation, in which the reinforcement of the tensile stress mechanisms that support the lateral ligaments of the ankle is considered important (Bleakley et al., 2019), which could be achieved by the activation of the Pero-L. Likewise, since the association between peroneal muscle fatigue and sprain is clear (Rodrigues, Soares & Tomazini, 2019), the use of selective instability devices such as the BB may be useful in the early stages in the prevention of ankle injuries, being part of strengthening programs. In contrast, BOSU or WB might be the selected devices for overall lower limb training, generating higher levels of muscle activation than PB or BB. To progress these programs in terms of intensity and degree of instability, it would be recommended to incorporate global instability devices in later phases, first PB and later BOSU and WB.

## Conclusions

According to the results obtained in healthy subjects, Pero-L activation seems to be similar on all the devices included and in the analyzed conditions. For the other muscles, each device produces a different level of activation. Thus, the BB elicits a similar electromyographic response (activation) of the Pero-L while causing less activation of the remaining muscles measured in this study. This could be useful in ankle injury prevention training programs, or perhaps in early stages of a rehabilitation process. PB would be a good device to progress in intensity, and finally, due to its high levels of activation for most muscles of the lower limb, the BOSU or WB should be included as instability training devices if the desire is for more functional training of the lower extremity rather than isolation of the stabilizing muscles of the ankle joint.

## Supplemental Information

10.7717/peerj.13317/supp-1 Supplemental Information 1 Raw data Variable gender: 1- Male, 2-Female

## Additional Information and Declarations

### Data Availability

The following information was supplied regarding data availability: The raw measurements are available in the Supplemental File.
